# Using gene expression from urine sediment to diagnose prostate cancer: development of a new multiplex mRNA urine test and validation of current biomarkers

**DOI:** 10.1186/s12885-016-2127-2

**Published:** 2016-02-09

**Authors:** Lourdes Mengual, Juan José Lozano, Mercedes Ingelmo-Torres, Laura Izquierdo, Mireia Musquera, María José Ribal, Antonio Alcaraz

**Affiliations:** Laboratory and Department of Urology, Hospital Clínic, Institut d’Investigacions Biomèdiques August Pi i Sunyer (IDIBAPS), Universitat de Barcelona, Barcelona, Spain; CIBERehd. Plataforma de Bioinformática, Centro de Investigación Biomédica en red de Enfermedades Hepáticas y Digestivas, Hospital Clínic, Institut d’Investigacions Biomèdiques August Pi i Sunyer (IDIBAPS), Universitat de Barcelona, Barcelona, Spain; Laboratory of Urology, Hospital Clínic, Centre de Recerca Biomèdica CELLEX, office B22, C/Casanova, 143, 08036 Barcelona, Spain

**Keywords:** Prostatic neoplasms, Gene expression, Urine, Diagnostic Techniques and Procedures, Tumor markers, Biological

## Abstract

**Background:**

Additional accurate non-invasive biomarkers are needed in the clinical setting to improve prostate cancer (PCa) diagnosis. Here we have developed a new and improved multiplex mRNA urine test to detect prostate cancer (PCa). Furthermore, we have validated the *PCA3* urinary transcript and some panels of urinary transcripts previously reported as useful diagnostic biomarkers for PCa in our cohort.

**Methods:**

Post-prostatic massage urine samples were prospectively collected from PCa patients and controls. Expression levels of 42 target genes selected from our previous studies and from the literature were studied in 224 post-prostatic massage urine sediments by quantitative PCR. Univariate logistic regression was used to identify individual PCa predictors. A variable selection method was used to develop a multiplex biomarker model. Discrimination was measured by ROC curve AUC for both, our model and the previously published biomarkers.

**Results:**

Seven of the 42 genes evaluated (*PCA3, ELF3, HIST1H2BG, MYO6, GALNT3, PHF12* and *GDF15*) were found to be independent predictors for discriminating patients with PCa from controls. We developed a four-gene expression signature (*HIST1H2BG, SPP1, ELF3* and *PCA3*) with a sensitivity of 77 % and a specificity of 67 % (AUC = 0.763) for discriminating between tumor and control urines. The accuracy of *PCA3* and previously reported panels of biomarkers is roughly maintained in our cohort.

**Conclusions:**

Our four-gene expression signature outperforms *PCA3* as well as previously reported panels of biomarkers to predict PCa risk. This study suggests that a urinary biomarker panel could improve PCa detection. However, the accuracy of the panels of urinary transcripts developed to date, including our signature, is not high enough to warrant using them routinely in a clinical setting.

**Electronic supplementary material:**

The online version of this article (doi:10.1186/s12885-016-2127-2) contains supplementary material, which is available to authorized users.

## Background

During the last two decades, prostate-specific antigen (PSA) has been extensively used for prostate cancer (PCa) screening, detection and follow-up. The routine use of PSA has been the subject of continued controversy owing to its limited specificity, which derives from the fact that elevated serum levels of PSA occur in a variety of non-neoplastic conditions such as prostatitis and benign prostate hyperplasia (BPH) [1]. Furthermore, up to 27 % of men with PSA in the normal range (≤ 4 ng/ml) suffer from PCa [2]. The current gold standard method for diagnosis of PCa in patients with elevated serum PSA is non-targeted transrectal ultrasound-guided needle biopsy, which fails to detect PCa in approximately 20–30 % of cases [3]. Therefore, there is a need for additional non-invasive and more specific markers of early PCa that will permit the stratification of patients according to their risk of developing PCa and thus identify men who will require prostate biopsy.

A great improvement in high-throughput gene expression techniques has yielded several promising molecular biomarkers for PCa detection. Prostatic cells can be collected in urine after an intensive prostatic massage. In 2003, Hessels et al. for the first time used the prostate cancer antigen 3 (*PCA3*) for the identification of PCa in urine sediments obtained after prostatic massage [4]. Since then, several studies have assessed the diagnostic performance of this marker (reviewed in [5, 6]) and other individual transcripts [7, 8]. However, taking into account the heterogeneity of PCa, several authors have searched for a multiplex detection system of biomarkers, which has proved to outperform the diagnostic value of the individual markers [9–12].

We have previously identified new putative mRNA markers for PCa diagnosis that can be extrapolated to post-prostatic massage (PPM) urine samples [13]. In the present study we aim to test several of those previously identified putative biomarkers in a large cohort of PPM-urine samples in order to develop an improved multiplex mRNA biomarker model for PCa diagnosis to be routinely used in the clinical setting. Furthermore, in our cohort we have validated the commercially available test based on urine *PCA3* expression as well as the best performing mRNA panels of biomarkers reported in the literature [9–12].

## Methods

### Patients and urine samples

Under Institutional Review Board approval (Hospital Clinic ethics committee) and patients’ informed consent, we prospectively collected 273 freshly voided urine samples from PCa patients and age matched controls between January 2009 and September 2012 at the Hospital Clínic of Barcelona. All patients underwent radical prostatectomy. The grade and stage of the tumours were determined according to Gleason criteria and TNM classification, respectively [14, 15]. Systematic prostate biopsy was performed to identify PCa patients included in the present study.

Voided urine samples (20 to 50 ml including the initial portion of the urine,) were collected following prostatic massage in sterile containers containing 2 ml of 0.5 M EDTA, pH 8.0. Urines were immediately stored at 4 °C and processed within the next 8 h. The samples were centrifuged at 1000xg for 10 min, at 4 °C. The cell pellets were re-suspended in 1 ml of TRIzol reagent (Invitrogen, Carlsbad, CA, USA) and frozen at −80 °C until RNA extraction.

### RNA extraction, cDNA synthesis and pre-amplification

RNAs from the urinary cell pellets were extracted using TRIzol reagent (Invitrogen, Carlsbad, CA, USA) according to the manufacturer’s instructions and quantified with a NanoDrop (NanoDrop Technologies, Wilmington, DE, USA).

cDNA was synthesized from 100 ng of total RNA using the High Capacity cDNA reverse transcription kit (Applied Biosystems, Foster City, CA USA; hereafter referred to as AB) following manufacturer’s instructions, except that the final volume of the reaction was 25 μl. A total of 1.25 μl of each cDNA sample, 2.5 μl of TaqMan PreAmp Master Mix kit 2X (AB) and 1.25 μl of pooled assay mix 0.2X containing 46 Gene Expression Assays (AB) were used for the multiplex pre-amplification of the target cDNAs following manufacturer’s instructions (AB). The 46 assays included in the pooled assay mix were selected from previous data from our group [13] and literature [10, 12, 16, 17] and contains 42 target genes and four endogenous controls; *B2M*, *GAPHDH, KLK2* and *KLK3* (Additional file 1: Table S1). Of note, 23 of the 42 target genes selected here were previously analyzed in urine samples by our group [13].

### Quantitative PCR using BioMark 48.48 Dynamic Arrays

A total of 2.25 μl of each pre-amplified cDNA was loaded into the Dynamic Array along with 0.25 μl of GE Sample Loading Reagent 20X (Fuidigm) and 2.5 μl of TaqMan Universal PCR Master Mix 2X (AB). For the assays, 2.5 μl of TaqMan® Gene Expression Assays 20X (AB) were combined with 2.5 μl of Assay Loading Reagent and were pipetted into the assay inputs. Reaction conditions were as follows: 50 °C for 2 min, 95 °C for 10 min, followed by 40 cycles of 95 °C for 15 s and 60 °C for 1 min. The real-time quantitative PCR (qPCR) experiments were performed on the BioMark instrument.

### Quantitative PCR data analysis

The real-time qPCR analysis software was used to obtain cycle quantification (Cq) values. Threshold was manually calculated for each gene. Since experimental errors such as inaccurate pipetting or contamination can result in amplification curves that look significantly different from a typical amplification curve, all amplification plots were checked both computationally and manually. Relative expression levels of target genes within a sample was expressed as ΔCq (ΔCq = Cq_endogenous control_-Cq_target gene_). We used as endogenous control the mean Cq value of *KLK2* and *KLK3*, which allowed us to normalize the prostate epithelial cell content in the collected urine sample [4]. Most of the studies seeking urinary transcripts for PCa diagnosis have used *KLK3* as a prostate-specific endogenous control [4, 18, 19]. In this study, to minimize the possibility of erroneous relative gene expression quantification, we also selected *KLK2* as a second prostate-specific endogenous control since its expression level is highly correlated with *KLK3* [20].

All 273 urine samples initially included in the study were positive for both housekeeping genes, the *B2MG* (*B2MG* mean Cq = 8.79; range 5.07–14.58) and *GAPDH* (*GAPDH* mean Cq = 10.85; range 7.6–16.17), indicating that all samples contained cells. Moreover, all samples were also positive for *KLK2* (*KLK2* mean Cq = 13.12; range 9.87–17.85) and for *KLK3* (*KLK3* mean Cq = 12.91; range 9.58–17.65) genes, indicating that all samples contained cells of prostate origin. Cq values for all other biomarkers are in the range for those of *KLK2* and *KLK3* (data not shown). All Cq values (except 2 cases in *B2MG* gene) fall in the optimal range of quantifiable Cq values in BioMark instrument (Cq = 6 to Cq = 23) [21]. Moreover, to assure the quality of the expression data obtained, low RNA quality samples were identified as outliers according to their average expression by the Mahalanobis Distance Quality Control (MDQC) method [22] and were excluded from the study. Fold change values were generated from the median expression of the genes from the BioMark 48.48 Dynamic Arrays in the groups compared.

### Statistical analysis

The association of each variable with final radical prostatectomy pathology results was analyzed by univariate logistic regression. Significance was defined as *p* values < 0.05.

All transcripts analyzed were subjected to variable selection using the lars function with method LASSO in the lars R statistical package (http://CRAN.R-project.org/package=lars) [23]. As all the samples were used for the model generation, the performance of the model may be over-optimized. To correct this bias, we further performed a leave-one-out cross-validation (LOOCV) and 100 randomisations with 5- fold cross-validation (5fCV) (http://CRAN.R-project.org/package=rms).

The optimal probability cutoff for the univariate study variables and logistic regression models (our model and those previously described in the literature [9–12]) was computed through a ROC analysis. To evaluate the performance of the models, we computed sensitivity (SN), specificity (SP), negative predictive value (NPV), positive predictive value (PPV) and overall error rates (ER) for the mRNA expression signature. Analysis of variance (ANOVA) of the Risk score probability versus three groups of PSA was done. Pairwise comparisons were made with Tukey’s HSD procedure. R-software was used for all calculations.

## Results

### Study population and informative rate

Among the 273 urine samples initially collected from 180 PCa patients and 93 control individuals, we excluded 29 urines from PCa patients (16 %) and 20 from controls (22 %) because they were flagged as low-quality samples when tested using MDQC method [22]. Thus, in total, the urine samples of 224 men, 151 with PCa and 73 controls were successfully analyzed (82 %). Table 1 shows characteristics and clinicopathological information for the 224 evaluable subjects. Only 10 patients with PSA levels > 4 were included as controls. Pathological reports from these patients confirmed the absence of malignity at the time of sample collection and they have not presented PCa during a mean follow-up of 45.6 months (range 19.5 to 78.9).

**Table 1:** Clinicohistopathologic features of the studied population

**Tumor urine samples**		
	**Mean ± SD (range)**	
Age (yr)	67.5 ± 7.9 (45–85)	
Gland weight (g)^a^	48.21 ± 22.88 (16–180)	
Serum PSA (ng/ml)^b^	13.76 ± 36.1 (0.94–365)	
	**Levels**	***N*** **patients (%)**
PSA (ng/ml)^b^	0–4	6 (4)
	4–10	96 (65)
	> 10	46 (31)
Gleason score^c^	< 7	69 (46)
	≥ 7	81 (54)
Stage^d^	T1	30 (27)
	T2	74 (65)
	T3	8 (7)
	T4	1 (1)
Treatment	RP	69 (46)
	RT	29 (19)
	CRT	23 (15)
	AS	23 (15)
	HT	7 (5)
**Control urine samples**		
	**Mean ± SD (range)**	
Age (yr)	67.2 ± 12 (21–97)	
Serum PSA (ng/ml)^e^	1.8 ± 1.06 (0.25–3.95)	
	***N*** **controls (%)**	
BPH/Prostatitis	35 (48)	
LUTS	18 (25)	
Lithiasis	5 (7)	
Urethral stenosis	4 (5)	
Others	11 (15)	

*Abbreviations*: *SD* Standard Deviation, *RP* Radical prostatectomy, *RT* Radiotherapy, *CRT* Cryotherapy, *AS* Active surveillance, *HT* Hormonal therapy, *BPH* Benign Prostate Hyperplasia, *LUTS* Low Urinary Tract Symptom

^a^Data available for 98 PCa patients; ^b^Data available for 148 PCa patients; ^c^Data available for 150 PCa patients; ^d^Data available for 113 PCa patients. Stage T1, only for those patients with no pathological stage available (Eg. RT, CRT, AS and HT); ^e^Data available for 65 controls

### Development of a new multiplex mRNA model

All 42 selected genes were first tested by univariate logistic regression analysis, with 7 genes (*PCA3, ELF3, HIST1H2BG, MYO6, GALNT3, PHF12* and *GDF15*) showing significant association for discriminating PCa patients from control individuals (Table 2 and Additional file 2: Table S2). Notably, no significant differences in *TMPRSS2-ERG* status between tumor (mean Cq = 13.54; range 10.28–18.21) and control (mean Cq = 13.88; range 10.28–18.71) urine samples were found. Differences in Cq values for *TMPRSS2-ERG* across the different Gleason stages (mean Cq = 13.54 for Gleason ≤ 6; mean Cq = 13.64 for Gleason = 7; mean Cq = 13.27 for Gleason ≥ 8) were not found either.

**Table 2:** Univariate logistic regression and ROC analyses of the biomarkers

Variable	Fold change	Univariate logistic regression analysis		ROC analysis
		OR (95 % CI)	*p* value	AUC (95 % CI)
*PCA3*	1.331	4.106 (7.534–2.237)	<0.01*	0.708 (0.742–0.675)
*ELF3*	−1.676	0.637 (0.818–0.496)	<0.01*	0.657 (0.693–0.621)
*MYO6*	−1.270	0.561 (0.826–0.381)	0.003*	0.622 (0.659–0.585)
*HIST1H2BG*	−1.243	0.609 (0.852–0.435)	0.004*	0.613 (0.65–0.575)
*GALNT3*	−1.064	0.556 (0.955–0.324)	0.033*	0.583 (0.622–0.545)
*PHF12*	−1.074	0.68 (0.982–0.471)	0.04*	0.567 (0.606–0.528)
*GDF15*	−1.240	0.681 (0.995–0.466)	0.047*	0.594 (0.632–0.556)
*PTOV1*	−1.110	0.615 (1.028–0.368)	0.063	0.592 (0.63–0.554)
*PSMA*	1.108	1.99 (4.153–0.954)	0.067	0.59 (0.628–0.552)
*SPINK1*	−1.156	0.74 (1.046–0.524)	0.089	0.572 (0.611–0.534)
*SOX4*	−1.148	0.663 (1.065–0.413)	0.089	0.571 (0.61–0.533)
*KLK12*	−1.142	0.639 (1.091–0.375)	0.101	0.577 (0.615–0.538)
*SLC44A5*	−1.090	0.646 (1.122–0.372)	0.121	0.57 (0.609–0.532)
*SPP1*	1.241	1.126 (1.335–0.95)	0.171	0.543 (0.582–0.504)
*DLX1*	−1.169	0.718 (1.16–0.444)	0.175	0.562 (0.601–0.523)
*CTHRC1*	−1.178	0.724 (1.157–0.453)	0.177	0.556 (0.595–0.518)
*TOX3*	−1.057	0.677 (1.198–0.383)	0.18	0.56 (0.599–0.522)
*TRPM4*	−1.130	0.69 (1.217–0.391)	0.2	0.554 (0.592–0.515)
*ELAVL2*	−1.068	0.734 (1.186–0.454)	0.207	0.554 (0.593–0.515)
*TWIST1*	−1.120	0.744 (1.19–0.466)	0.217	0.558 (0.6–0.52)

*Abbreviations*: *OD* odds ratio, *95 % CI* 95 % confidence interval, *AUC* Area Under the Curve

*Statistically significant (*p* < 0.05)

Note: Only biomarkers presenting a *p* value < 0.25 are listed. Univariate logistic regression and ROC analysis for all biomarkers is shown in Additional file 2: Table S2

**Fig. 1:**
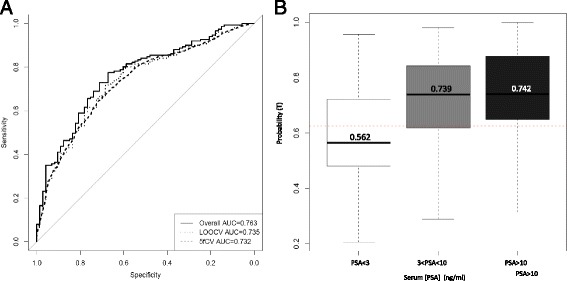
Diagnostic performance of the four –gene expression signature. **a** ROC analysis based on the predicted probabilities derived from the four-gene model. **b** Probabilistic sensitivity analysis of the signature according to serum PSA levels

To evaluate the performance of individual markers for diagnosing PCa, we performed a ROC analysis (Table 2). Then, individual biomarkers were subjected to variable selection to develop a multiplex model that could improve performance over single biomarkers. This analysis resulted in a final selection of a four-gene model that contains *HIST1H2BG, SPP1, ELF3* and *PCA3*. The four gene model outperformed single genes and previously reported models in the literature in detecting PCa in urinary sediments (SN = 77 %; SP = 67 %; PPV = 83 %; NPV = 58 %; ER = 26 %; AUC = 0.763). After applying LOOCV analysis to the four-gene model, we obtained a SN of 79 % for discriminating between tumor and control urines with a SP of 60 % (PPV = 80 %; NPP = 58 %; ER = 27 %; AUC 0.735). By using 5fCV analysis, we found a SN of 72.52 % for discriminating between tumor and control urines with a SP of 64.83 % (PPV = 80.86 %; NPV = 53.5 %; ER = 30 %; AUC 0.732) (Fig. 1a). To note, the four-gene model also performs well in the diagnostic PSA gray-zone (PSA 3–10 ng/ml) yielding a SN of 79 % for discriminating between tumor urines from patients with PSA serum values between 3 and 10 ng/ml and control urines, with a SP of 59 % (PPV = 72 %; NPP = 68 %; ER = 29 %; *p* < 0.001) (Fig. 1b).

### Evaluation of previously reported diagnostic biomarkers of urinary transcripts in our cohort

First, we evaluated the *PCA3* marker (TaqMan PCR test for *PCA3*) as a single marker. Univariate logistic regression analysis showed that expression of *PCA3* was a significant discriminator of PCa from control individuals (*p* < 0.01). *PCA3* alone achieved an overall SN of 49 % and a SP of 85 % (AUC = 0.708) to discriminate controls from PCa urines (Table 2 and Additional file 2: Table S2). Then, we evaluated in our cohort some of the most potentially promising PCa diagnostic panels of urinary transcripts reported in the literature, to validate their performance in an independent set. Table 3 summarizes the diagnostic performance of the biomarkers panels in our case-control setting in comparison to the results obtained in the original studies. As shown, all the biomarker combinations roughly maintain their performance when tested in an independent set, the combination described by Laxman et al. (2008) having the best performance [10].

**Table 3:** Diagnostic performance for PCa of the most significant urine-gene expression signatures containing PCA3 gene, reported originally and validated in our cohort

Study	Biomarkers	Initial performance reported				Performance validation (our cohort)			
		n total (T/C)	SN (%)	SP (%)	AUC	n total (T/C)	SN (%)	SP (%)	AUC
Hessels et al., 2003 [4]	*PCA3*	108 (24/84)	67	83	0.717	224 (151/73)	49	85	0.708
Hessels et al., 2007 [9]	*PCA3, TMPRSS2:ERG*	108 (78/30)	73	52	-	224 (151/73)	48	86	0.708
Laxman et al., 2008 [10]	*PCA3, TMPRSS2:ERG, GOLPH2, SPINK1*	234 (138/96)	66	76	0.758	224 (151/73)	72	64	0.719
Ouyang et al., 2009 [11]	*PCA3, AMACAR*	92 (43/49)	81	53	-	224 (151/73)	48	85	0.707
Rigau et al., 2010 [12]	*PCA3, PSGR*	215 (73/142)	77	60	0.73	224 (151/73)	62	71	0.708
Present study, 2015	*HIST1H2BG, SPP1, ELF3* and *PCA3*	224 (151/73)	77	83	0.763	224 (151/73)	77	83	0.763

*Abbreviations*: *T* Tumors, *C* Controls, *SN* Sensitivity, *SP* Specificity, *AUC* Area Under the Curve

## Discussion

Currently, PSA is considered the most valuable tool in the early detection, staging and monitoring of PCa. However, as mentioned in the introduction, PSA has several limitations as a PCa diagnostic biomarker, especially in deciding the necessity of a prostate biopsy. Actually, PCa is detected in only about a third of patients with elevated serum PSA who undergo random prostate biopsy. Repeated biopsies reveal the presence of PCa in another 10–35 % of the cases [24]. Not only economic aspects but also anxiety, discomfort, and sometimes severe complications are associated with prostate biopsies. Therefore, the development of a non-invasive diagnostic tool for the early detection and screening of PCa as well as to increase the probability of detecting PCa at repeat biopsy, reducing the number of unnecessary biopsies, is needed in urological practice. Detection of aberrantly expressed transcripts in PCa cells shed into the urine after prostatic massage are promising biomarkers for the development of a reliable non-invasive PCa diagnostic method. In fact, several promising RNA-based urine PCa biomarkers are described in the literature, but only the *PCA3* assay (Progensa) is approved by the FDA and currently is the only molecular diagnostic assay for PCa commercially available. However, *PCA3* is not routinely used in the clinical setting mainly because clinicians feel that the increase in accuracy over serum PSA testing is not significant enough to warrant a biopsy. Furthermore, since PCa is a heterogeneous disease, it is reasonable that a combination of markers outperforms single marker detection. In this regard, several authors have described combinations of RNA-markers in urine samples but to our knowledge, none of them, except one [25], has been externally validated nor is currently used in the clinical setting. In the present work, we have developed a four-gene panel that outperforms those previously described in the literature. In addition, in our cohort we have validated *PCA3* as well as the most promising panels of biomarkers described.

From our analysis, we have been able to identify six new candidates that independently predict PCa in PPM-urine samples, besides *PCA3*. This has been possible since we have explored target genes selected from previous PCa microarray data [13, 17] instead of analyzing only previously described prostate related biomarkers. Actually, all target genes explored were used to develop the four-gene set model that contains the previously described *PCA3* gene and three new biomarkers: *HIST1H2BG, SPP1* and *ELF3*. This model outperforms individual biomarkers and previously reported models in the literature. Although LOOCV indicates a certain degree of overfitting, all data obtained after cross validation corroborate the SN and SP for the final model. Moreover, the model performs well in the diagnostic PSA gray-zone (PSA 3–10 ng/ml) where a reduction in the number of unnecessary biopsies is necessary.

Notably, the three new biomarkers of the model had been previously associated with PCa. Alterations in expression of histone *HIST1H2BG* were associated with biochemical recurrence in PCa patients after radical prostatectomy [26]. The transcription factor *ELF3* (E74-like factor 3), that acts as a negative modulator of androgen receptor transcriptional activity, was found underexpressed in PCa [27], according to our results. On the other hand, *SPP1* (secreted phosphoprotein 1) encodes the protein osteopontin (OPN). Both, OPN RNA and protein have been found overexpressed in a number of human tumor types, including PCa [28]. In some cases, OPN overexpression has been shown to be associated directly with poor patient prognosis or with other indicators of poor prognosis. Thus, OPN has a dual interest, as a biomarker of malignancy as well as a candidate for testing as a poor prognostic factor. Even though in the present study we did not achieve statistical significance for *SPP1*, the addition of this gene to the model improved the AUC from 0.740 (*HIST1H2BG, PCA3* and *ELF3*) to 0.763 (*SPP1*, *HIST1H2BG, PCA3* and *ELF3*), indicating that effectively its expression adds information to the model.

The present study confirms that *PCA3* can successfully discriminate PCa from controls in randomly selected patients with variable PSA levels (PSA = 0.94–365 ng/ml) [29, 30]. A limitation of most studies based on urinary biomarkers is that the negative PCa patient group consists of patients who have undergone prostate biopsy for suspected PCa with a negative result, but in fact, 20–30 % of such patients will be diagnosed with PCa at a later date [3]. To overcome this limitation, our control group consisted of patients without suspected PCa (PSA < 4.0 ng/ml), thus minimizing the risk of including subjects with PCa in the control group. Moreover, there is no uniform methodological protocol for urinary transcript quantification in the reported studies. For instance, some studies use a multiplex cDNA preamplification step before qPCR transcript quantification [16, 31], while others use a Whole Transcriptome Amplification [10, 32] or even in some studies cDNA is not preamplifed [11]. Also different gene expression normalization methods are used [4, 11, 16, 18, 31]. Thus, it is notable that despite this methodological heterogeneity and the inherent limitations of the sample source (PPM-urine contains different cell types, including renal tubular cells, urothelial cells, prostate cells, etc.… and the proportion of prostate tumor cells in each subject is different), we and the vast majority of the groups identify *PCA3* as an independent predictor for PCa diagnosis, making it the most reliable individual biomarker to date.

However, combining urinary biomarkers in a panel has shown higher diagnostic accuracy than *PCA3* alone. Regarding this, we have been able to validate some of the previously reported panels of biomarkers [9–12] in our cohort and to develop a new urinary panel of biomarkers that improves serum PSA and previously reported panels of biomarkers. On the contrary, we could not validate differences between control and cancer population for the *TMPRSS2-ERG* status. This is in all probability due to the methodological approach used here, since others using the same methodology as us (RT-qPCR using the same gene expression assay as us; Hs03063375_ft ) to evaluate *TMPRSS2-ERG* status also did not find differences between cancer and control urines [33] while other authors using Southern blot [9] or transcription-mediated amplification [32] were able to find such differences.

Of concern, neither the FDA approved *PCA*3 test alone, or in combination with other biomarkers, is being routinely used in the clinical setting. This is most likely because the addition of urine biomarkers to the current clinical diagnostic tools only shows a limited improvement in the PCa diagnosis accuracy and does not provide sufficient value to affect biopsy decision making. In fact, recently the Evaluation of Genomic Applications in Practice and Prevention Working Group (EWG) has found insufficient evidence to recommend *PCA3* testing not only for deciding to conduct initial biopsies for PCa at risk men (e.g. previously elevated PSA test or suspicious digital rectal examination) but also for deciding when to rebiopsy previously biopsy-negative patients for PCa. Furthermore, the EWG did not find convincing evidence to recommend *PCA3* testing in men with PCa positive-biopsies to determine whether the disease is indolent or aggressive, in order to develop an optimal treatment plan [34]. Thus, even though many efforts have been made in the last decade to identify urine biomarkers that determine men at high risk of PCa and whether the disease is indolent or aggressive in men with PCa, the results do not seem convincing for clinicians.

We acknowledge that our study has several limitations. First it resides in the relatively low sample size of the studied cohort. This was because 18 % of urine samples collected could not be evaluated (informative specimen rate of 82 %). Although some improvements in the methodological process would be desirable to decrease the percentage of fails, this percentage is in the range of those described by other authors who quantify gene expression in PPM urine samples (informative specimen rates 56 to 92 %) [10–12, 16, 30, 31]. However, sample collection can be repeated if necessary. It could also be argued that we arbitrarily selected the 42 target genes, while the list of differentially expressed genes in PCa is much larger. In this regard, we have tried to include the biomarkers according to previous studies, as being either detectable in urine or appropriate for combined models, and genes highly differentially expressed in PCa tissue samples. We are also aware that we should test the performance of our four-gene expression signature in a real clinical scenario by analyzing patients who undergo prostate biopsy for suspected PCa, even though this study will have the limitation of false negative biopsies, which account for 20–30 % of men at risk of PCa [3]. Lastly, future validation studies are needed to further improve the performance of this test by examination of larger and independent cohorts.

## Conclusions

We report a four-gene expression signature with higher diagnostic accuracy than *PCA3*, the only non-invasive commercially available urinary biomarker, to predict individuals at risk of PCa. Moreover, our four-gene expression signature outperforms previously reported panels of biomarkers for PCa detection. Taken together, these results suggest that new biomarkers can be successfully combined with *PCA3*, resulting in improvements in PCa detection. However, further sources of new non-invasive biomarkers that enable physicians to accurately predict any PCa at initial prostate biopsy and aggressive PCa should be explored.

## Additional files

Additional file 1: Table S1. Commercial Gene Expression Assays from Life Technologies used in this study. Target exons for transcript detection as well as amplicon length for each trasncrip are shown. (DOCX 18 kb) Additional file 2: Table S2. Univariate logistic regression and ROC analyses of the biomarkers. (DOCX 19 kb)

## Abbreviations

5fCV: 5-fold cross-validation
AUC: area under curve
BPH: benign prostate hyperplasia
CI: confidence interval
Cq: cycle quantification
ER: error rate
LOOCV: leave-one-out cross-validation
NPV: negative predictive value
OD: odds ratio
PCa: prostate cancer
PPM: post-prostatic massage
PPV: positive predictive value
PSA: Prostate-specific antigen
qPCR: quantitative PCR
ROC: receiver operator characteristic
SD: standard deviation
SN: sensitivity
SP: specificity
