# Supplementary material for: Using gene expression from urine sediment to diagnose prostate cancer: development of a new multiplex mRNA urine test and validation of current biomarkers
Source: BMC Cancer. 2016 Feb 9;16:76. doi: 10.1186/s12885-016-2127-2 (PMC4746764; doi:10.1186/s12885-016-2127-2)
Supplement: Additional file 1: Table S1. — Commercial Gene Expression Assays from Life Technologies used in this study. Target exons for transcript detection as well as amplicon length for each trasncrip are shown. (DOCX 18 kb) [file 12885_2016_2127_MOESM1_ESM.docx]

**Table S1.** Commercial Gene Expression Assays from Life Technologies used in this study. Target exons for transcript detection as well as amplicon length for each trasncrip are shown.

| **Gene symbol** | **Gene expression assay** | **RefSeq** | **Exon Boundary** | **Assay Location** | **Amplicon Length** |
| --- | --- | --- | --- | --- | --- |
| *ABL1* | Hs00245445_m1 | NM_005157.4 | 3-4 | 549 | 91 |
| *AMACR** | Hs00204885_m1 | NM_001167595.1 | 3-4 | 652 | 141 |
| *B2M* | Hs99999907_m1 | NM_004048.2 | 2-3 | 409 | 75 |
| *CDK1* | Hs00938778_m1 | NM_001786.4 | 4-5 | 456 | 77 |
| *CRISP3* | Hs00195988_m1 | NM_001190986.1 | 7-8 | 746 | 111 |
| *CTHRC1* | Hs00298917_m1 | NM_001256099.1 | 2-3 | 453 | 89 |
| *DLX1** | Hs00698288_m1 | NM_178120.4 | 1-2 | 514 | 95 |
| *ECT2* | Hs00216455_m1 | NM_001258315.1 | 24-25 | 2851 | 94 |
| *ELAVL2** | Hs00270011_m1 | NM_001171195.1 | 4-5 | 741 | 118 |
| *ELF3* | Hs00963881_m1 | NM_001114309.1 | 8-9 | 1263 | 64 |
| *ERG** | Hs00171666_m1 | NM_001136154.1 | 4-5 | 529 | 60 |
| *ETV1** | Hs00231877_m1 | NM_001163147.1 | 7-8 | 1096 | 75 |
| *GALNT3** | Hs00237084_m1 | NM_004482.3 | 2-3 | 897 | 66 |
| *GAPDH* | Hs99999905_m1 | NM_002046.4 | 3-3 | 229 | 122 |
| *GDF15* | Hs00171132_m1 | NM_004864.2 | 1-2 | 313 | 78 |
| *GOLM1** | Hs00213061_m1 | NM_016548.3 | 3-4 | 518 | 88 |
| *HIST1H2BG* | Hs00374317_s1 | NM_003518.3 | 1-1 | 392 | 85 |
| *HOXC6** | Hs00171690_m1 | NM_004503.3 | 1-2 | 515 | 87 |
| *INSM1* | Hs00357871_s1 | NM_002196.2 | 1-1 | 845 | 72 |
| *KLK12** | Hs00377603_m1 | NM_019598.2 | 5-6 | 707 | 108 |
| *KLK2* | Hs00428384_g1 | NM_001002231.2 | 3-4 | 529 | 145 |
| *KLK3* | Hs00426859_g1 | NM_001030047.1 | 2-3 | 243 | 153 |
| *MUC12** | Hs00415869_m1 | NM_001164462.1 | 9-10 | 15798 | 88 |
| *MYO6** | Hs00192265_m1 | NM_004999.3 | 19 - 20 | 2264 | 110 |
| *NUSAP1* | Hs01006195_m1 | NM_001243142.1 | 7 - 8 | 1102 | 87 |
| *PCA3** | Hs01371939_g1 | NR_015342.1 | 2-3 | 308 | 52 |
| *PCSK6** | Hs00159844_m1 | NM_002570.3 | 9 - 10 | 1627 | 82 |
| *PDK4** | Hs00176875_m1 | NM_002612.3 | 2 - 3 | 590 | 85 |
| *PHF12** | Hs00604572_m1 | NM_001033561.1 | 5 - 6 | 1373 | 71 |
| *PSGR (OR51E2)* | Hs04231197_m1 | NM_030774.3 | 1 - 2 | 199 | 61 |
| *PSMA (FOLH1)** | Hs00379515_m1 | NM_001014986.1 | 4 - 5 | 769 | 110 |
| *PTOV1* | Hs00363189_g1 | NM_017432.3 | 7 - 8 | 977 | 67 |
| *PVT1** | Hs00413039_m1 | M25803.1 | - | 175 | 111 |
| *RRM2* | Hs00357247_g1 | NM_001034.3 | 1 - 2 | 163 | 79 |
| *SIM2* | Hs00894178_m1 | NM_005069.3 | 9 - 10 | 1227 | 69 |
| *SLC44A5** | Hs00380535_m1 | NM_001130058.1 | 14 - 15 | 1228 | 106 |
| *SOX4* | Hs00268388_s1 | NM_003107.2 | 1 - 1 | 797 | 143 |
| *SPINK1** | Hs00162154_m1 | NM_003122.3 | 2 - 3 | 208 | 85 |
| *SPP1* | Hs00959010_m1 | NM_000582.2 | 5 - 6 | 657 | 84 |
| *TFF3** | Hs00173625_m1 | NM_003226.3 | 1 - 2 | 304 | 98 |
| *TMPRSS2-ERG fusion** | Hs03063375_ft | DQ204772.1 | - | 49 | 106 |
| *TOP2A* | Hs00172214_m1 | NM_001067.3 | 10 - 11 | 1367 | 125 |
| *TOX3** | Hs00300355_m1 | NM_001080430.2 | 6 - 7 | 1157 | 101 |
| *TRPM4* | Hs00214167_m1 | NM_001195227.1 | 12 - 13 | 1852 | 60 |
| *TWIST1** | Hs00361186_m1 | NM_000474.3 | 1 - 2 | 1004 | 115 |
| *UBE2C* | Hs00964100_g1 | NM_001281741.1 | 4 - 5 | 464 | 64 |

*Genes analyzed in our previous study [13].
