# Supplementary material for: Using gene expression from urine sediment to diagnose prostate cancer: development of a new multiplex mRNA urine test and validation of current biomarkers
Source: BMC Cancer. 2016 Feb 9;16:76. doi: 10.1186/s12885-016-2127-2 (PMC4746764; doi:10.1186/s12885-016-2127-2)
Supplement: Additional file 2: Table S2. — Univariate logistic regression and ROC analyses of the biomarkers. (DOCX 19 kb) [file 12885_2016_2127_MOESM2_ESM.docx]

**Table S2.** Univariate logistic regression and ROC analyses of the biomarkers

| **Variable** | **Fold change** | **Univariate logistic regression analysis** | | | **ROC analysis** |
| --- | --- | --- | --- | --- | --- |
|  |  | **OR (95% CI)** | **p value** | **FDR** | **[AUC (95% CI)]** |
| *PCA3* | 1.331 | 4.106 (7.534-2.237) | <0.01* | <0.01* | 0.708 (0.742-0.675) |
| *ELF3* | -1.676 | 0.637 (0.818-0.496) | <0.01* | <0.01* | 0.657 (0.693-0.621) |
| *MYO6* | -1.270 | 0.561 (0.826-0.381) | 0.003* | 0,043* | 0.622 (0.659-0.585) |
| *HIST1H2BG* | -1.243 | 0.609 (0.852-0.435) | 0.004* | 0,043* | 0.613 (0.65-0.575) |
| *GALNT3* | -1.064 | 0.556 (0.955-0.324) | 0.033* | 0,296 | 0.583 (0.622-0.545) |
| *PHF12* | -1.074 | 0.68 (0.982-0.471) | 0.04* | 0,296 | 0.567 (0.606-0.528) |
| *GDF15* | -1.240 | 0.681 (0.995-0.466) | 0.047* | 0,301 | 0.594 (0.632-0.556) |
| *PTOV1* | -1.110 | 0.615 (1.028-0.368) | 0.063 | 0,334 | 0.592 (0.63-0.554) |
| *PSMA* | 1.108 | 1.99 (4.153-0.954) | 0.067 | 0,334 | 0.59 (0.628-0.552) |
| *SPINK1* | -1.156 | 0.74 (1.046-0.524) | 0.089 | 0,364 | 0.572 (0.611-0.534) |
| *SOX4* | -1.148 | 0.663 (1.065-0.413) | 0.089 | 0,364 | 0.571 (0.61-0.533) |
| *KLK12* | -1.142 | 0.639 (1.091-0.375) | 0.101 | 0,377 | 0.577 (0.615-0.538) |
| *SLC44A5* | -1.090 | 0.646 (1.122-0.372) | 0.121 | 0,398 | 0.57 (0.609-0.532) |
| *SPP1* | 1.241 | 1.126 (1.335-0.95) | 0.171 | 0,451 | 0.543 (0.582-0.504) |
| *DLX1* | -1.169 | 0.718 (1.16-0.444) | 0.175 | 0,451 | 0.562 (0.601-0.523) |
| *CTHRC1* | -1.178 | 0.724 (1.157-0.453) | 0.177 | 0,451 | 0.556 (0.595-0.518) |
| *TOX3* | -1.057 | 0.677 (1.198-0.383) | 0.18 | 0,451 | 0.56 (0.599-0.522) |
| *TRPM4* | -1.130 | 0.69 (1.217-0.391) | 0.2 | 0,465 | 0.554 (0.592-0.515) |
| *ELAVL2* | -1.068 | 0.734 (1.186-0.454) | 0.207 | 0,465 | 0.554 (0.593-0.515) |
| *TWIST1* | -1.120 | 0.744 (1.19-0.466) | 0.217 | 0,466 | 0.558 (0.6-0.52) |
| *INSM1* | -1.016 | 1.226 (1.801-0.835) | 0.299 | 0,608 | 0.516 (0.556-0.477) |
| *HOXC6* | -1.078 | 0.749 (1.312-0.427) | 0.312 | 0,608 | 0.546 (0.585-0.507) |
| *CRISP3* | -10.943 | 0.915 (1.095-0.764) | 0.333 | 0,608 | 0.524 (0.564-0.485) |
| *PVT1* | -1.047 | 0.765 (1.322-0.443) | 0.338 | 0,608 | 0.541 (0.58-0.502) |
| *CDK1* | -1.038 | 0.786 (1.364-0.453) | 0.392 | 0,626 | 0.537 (0.576-0.498) |
| *PDK4* | -1.014 | 0.7924 (1.358-0.462) | 0.398 | 0,626 | 0.549 (0.588-0.51) |
| *TMPRSS2:ERG* | -1.041 | 0.8 (1.351-0.474) | 0.404 | 0,626 | 0.535 (0.574-0.496) |
| *PSGR* | 1.066 | 1.305 (2.52-0.676) | 0.427 | 0,640 | 0.546 (0.585-0.507) |
| *MUC12* | -1.063 | 0.832 (1.505-0.46) | 0.543 | 0,765 | 0.536 (0.578-0.499) |
| *ERG* | -1.047 | 0.849 (1.466-0.491) | 0.556 | 0,765 | 0.532 (0.571-0.493) |
| *ECT2* | -1.017 | 0.8441 (1.498-0.476) | 0.563 | 0,765 | 0.525 (0.564-0.486) |
| *TFF3* | -1.025 | 0.843 (1.538-0.462) | 0.578 | 0,765 | 0.523 (0.563-0.484) |
| *AMACR* | -1.004 | 1.187 (2.323-0.607) | 0.616 | 0,792 | 0.506 (0.546-0.467) |
| *UBE2C* | 1.036 | 1.093 (1.688-0.708) | 0.687 | 0,836 | 0.503 (0.543-0.463) |
| *ETV1* | 1.094 | 0.91 (1.557-0.532) | 0.731 | 0,860 | 0.522 (0.561-0.483) |
| *NUSAP1* | 1.038 | 0.935 (1.437-0.608) | 0.759 | 0,860 | 0.509 (0.549-0.47) |
| *SIM2* | 1.02 | 0.899 (1.804-0.448) | 0.764 | 0,860 | 0.5 (0.54-0.461) |
| *GOLM1* | -1.021 | 0.974 (1.641-0.579) | 0.922 | 0,976 | 0.511 (0.55-0.471) |
| *TOP2A* | 1.03 | 0.979 (1.741-0.551) | 0.942 | 0,976 | 0.506 (0.545-0.466) |
| *ABL1* | -1.017 | 0.98 (1.761-0.544) | 0.944 | 0,976 | 0.512 (0.551-0.472) |
| *PCSK6* | -1.032 | 0.985 (1.792-0.542) | 0.962 | 0,976 | 0.512 (0.551-0.472) |
| *RRM2* | 1.026 | 1.009 (1.765-0.576) | 0.976 | 0,976 | 0.493 (0.533-0.453) |

Abbreviations: OD, odds ratio; 95% CI, 95% confidence interval; FDR, False Discovery Rate; AUC, Area Under the Curve.

* Statistically significant (p<0.05; FDR>0.1).
